# CD133^+^CXCR4^+ ^colon cancer cells exhibit metastatic potential and predict poor prognosis of patients

**DOI:** 10.1186/1741-7015-10-85

**Published:** 2012-08-07

**Authors:** Shan-shan Zhang, Zhi-peng Han, Ying-ying Jing, Shuang-fen Tao, Tie-jun Li, Hao Wang, Yang Wang, Rong Li, Yang Yang, Xue Zhao, Xiao-dong Xu, En-da Yu, Yao-cheng Rui, Hou-jia Liu, Li Zhang, Li-xin Wei

**Affiliations:** 1 Tumor Immunology and Gene Therapy Center, Eastern Hepatobiliary Surgery Hospital, Second Military Medical University, 225 Changhai Road, Shanghai 200438, China; 2 Department of Pharmacology, Second Military Medical University, 325 Guohe Road, Shanghai 200433, China; 3 Changhai Hospital, Second Military Medical University, 168 Changhai Road, Shanghai 200433, China

**Keywords:** colorectal cancer, cancer stem cell, CXCR4, epithelial-mesenchymal transition, liver metastasis

## Abstract

**Background:**

Colorectal cancer (CRC), which frequently metastasizes to the liver, is one of the three leading causes of cancer-related deaths worldwide. Growing evidence suggests that a subset of cells exists among cancer stem cells. This distinct subpopulation is thought to contribute to liver metastasis; however, it has not been fully explored in CRC yet.

**Methods:**

Flow cytometry analysis was performed to detect distinct subsets with CD133 and CXCR4 markers in human primary and metastatic CRC tissues. The 'stemness' and metastatic capacities of different subpopulations derived from the colon cancer cell line HCT116 were compared *in vitro *and *in vivo*. The roles of epithelial-mesenchymal transition (EMT) and stromal-cell derived factor-1 (SDF-1) in the metastatic process were also investigated. A survival curve was used to explore the correlation between the content of CD133^+^CXCR4^+ ^cancer cells and patient survival.

**Results:**

In human specimens, the content of CD133^+^CXCR4^+ ^cells was higher in liver metastases than in primary colorectal tumors. Clonogenic and tumorigenic cells were restricted to CD133^+ ^cells in the HCT116 cell line, with CXCR4 expression having no impact on the 'stemness' properties. We found that CD133^+^CXCR4^+ ^cancer cells had a high metastatic capacity *in vitro *and *in vivo*. Compared with CD133^+^CXCR4^- ^cells, CD133^+^CXCR4^+ ^cancer cells experienced EMT, which contributed partly to their metastatic phenotype. We then determined that SDF-1/CXCL12 treatment could further induce EMT in CD133^+^CXCR4^+ ^cancer cells and enhance their invasive behavior, while this could not be observed in CD133^+^CXCR4^- ^cancer cells. Blocking SDF-1/CXCR4 interaction with a CXCR4 antagonist, AMD3100 (1,10-[1,4-phenylenebis(methylene)]bis-1,4,8,11 -tetraazacyclotetradecane octahydrochloride), inhibited metastatic tumor growth in a mouse hepatic metastasis model. Finally, a high percentage of CD133^+^CXCR4^+ ^cells in human primary CRC was associated with a reduced two-year survival rate.

**Conclusions:**

Strategies targeting the SDF-1/CXCR4 interaction may have important clinical applications in the suppression of colon cancer metastasis. Further investigations on how high expression of CXCR4 and EMT occur in this identified cancer stem cell subset are warranted to provide insights into our understanding of tumor biology.

## Background

Colorectal cancer (CRC) is among the three leading causes of cancer-related deaths worldwide. Nearly 50% of patients with CRC develop liver metastases synchronously or metachronously, and in advanced disease the mortality of CRC is principally attributable to the development of hepatic metastases [1,2]. Therefore, it is important to uncover the biological mechanisms underlying liver metastasis of CRC and accelerate the development of new treatment strategies.

Cancer stem cells (CSCs) have moved to the center stage in cancer research in recent years and have been viewed as the origin of cancer formation, development and metastasis. CSCs possess the ability to self-renew and to differentiate into phenotypically diverse progeny, a subpopulation within a tumor that could also be labeled tumor-initiating cells [3-5]. Investigation into hematopoietic stem cells has led the way for CSC research [6], and has been followed by studies showing the existence of CSCs in various types of tumors, including colon cancer [7-12]. Recently, Brabletz and colleagues proposed a concept that CSCs may represent a heterogeneous population consisting of two forms of CSCs during tumor progression, namely stationary and migrating CSCs. The latter is a small subpopulation that combines the two most decisive traits, stemness and mobility, and thus holds important clues for the further understanding of malignant progression [13].

Recent studies have highlighted the role of chemokines in cancer metastasis. According to the signaling/homing theory, target organs produce and release specific chemokines and attract nearby or distant cancer cells bearing corresponding receptors [14]. These studies have suggested that the stromal cell-derived factor-1 (SDF-1/CXCR4) axis plays a key role in tumor invasiveness leading to local progression and tumor metastasis in lung, pancreatic, and breast cancers, as well as CRCs [15-20]. Hermann *et al*. found that in human pancreatic cancers, a distinct subpopulation of CD133^+^CXCR4^+ ^CSCs was identified that determines the metastatic phenotype of the individual tumor. Depletion of this specific stem cell population virtually abrogated the tumor metastatic phenotype without affecting their tumorigenic potential [21]. However, the existence of a migrating subpopulation expressing CD133 and CXCR4 has not been reported in CRC.

The acquisition of the mesenchymal phenotype by epithelial cells, known as the epithelial-mesenchymal transition (EMT), is a key process that is required during embryonic development. Epithelial cells have tight cell-cell contact *via *various junctions, which only allow limited movement of epithelial cells. In contrast, with an elongated spindle shape, mesenchymal cells interact with neighboring cells to a limited extent (and only at focal points) and have increased motility [22,23]. EMT is associated with cancer cell migration and metastasis, and cancer cells acquire a more aggressive phenotype *via *EMT, indicating that it is a crucial event in malignancy [24-27]. Some studies have reported a correlation between CSCs and EMT [27-30]. We hypothesized that EMT plays an essential role in endowing migratory CSCs with metastatic capacity. In this study, we have provided evidence for the existence of a distinct migrating CSC subpopulation of CD133^+^CXCR4^+ ^cells in human CRC specimens as well as in the human colon cancer cell line, HCT116. We found that EMT and the SDF-1/CXCR4 axis are involved in the metastatic process.

## Methods

### Tissue samples

Primary CRC and metastatic liver cancer tissue samples were obtained from 29 patients undergoing surgical resection of primary CRC and/or liver metastasis at the Department of Surgery, Changhai Hospital and Eastern Hepatobiliary Surgery Hospital of the Second Military Medical University from February 2007 to May 2008. After resection, patients were followed up every three months. Sections were reviewed by two experienced pathologists to verify the histologic assessment. All the specimens were adenocarcinoma. Prior informed consent was obtained and the study protocol was approved by the Ethics Committee of the Second Military Medical University.

### Cell culture and animals

The human colon cancer cell line, HCT116, was maintained in McCoy's 5A Medium (GIBCO, Invitrogen, Carlsbad, CA, USA) supplemented with 10% fetal bovine serum (FBS; GIBCO, Invitrogen), 100 units/ml penicillin and 100 mg/ml streptomycin in a humidified incubator under 95% air and 5% CO_2 _at 37°C.

Male nude mice (BALB/c strain), six to eight weeks old, were purchased from the Shanghai Experimental Animal Center of the Chinese Academy of Sciences (Shanghai, China). Mice in this study were housed under pathogen-free conditions, and all procedures were performed in accordance with the institutional animal welfare guidelines of the Second Military Medical University.

### Flow cytometry and FACS

Fresh specimens from primary CRC, hepatic metastatic cancer and their corresponding normal tissues were transferred to a petri dish, where the tissue was gently minced and filtered (100 mm) to remove large aggregates. This was followed by incubation for 45 minutes at 37°C in 50 ml of Hank's balanced salt solution containing 0.05% collagenase, with continuous stirring. DNAase (0.5 mg) in 1.0 ml of PBS was added 20 to 40 minutes after this incubation period. The cell suspension was filtered (40 mm), and non-parenchymal cells were separated by discontinuous density gradients of Percoll (Pharmacia Biotech, Piscataway, NJ, USA) at 1.044 g/ml and 1.07 g/ml. The final cell suspension was washed twice, and CD133 (Miltenyi Biotech, Bergisch Gladbach, Germany) and/or CXCR4 antibody (eBioscience, San Diego, CA, USA) was added and incubated at 4°C for 20 minutes before washing. Stained cells were analyzed using flow cytometry.

The CD133^+^CXCR4^+ ^cancer cell content determined by flow cytometry was utilized to investigate the correlation between CD133^+^CXCR4^+ ^cancer cells and clinical characteristics and two-year survival. Suspensions of HCT-116 cells (10^7^/ml) were sorted according to the expression of CD133 and CXCR4 with a fluorescence activated cell sorting system (FACS, Becton Dickinson, San Jose, CA, USA) following multicolor staining as described for flow cytometric analyses. Separated subpopulations were reanalyzed for purity and then used in subsequent experiments.

### Standard tail vein metastatic assay

Tumor cells (5 × 10^5^) were injected into the lateral tail vein using a 27-gauge needle, more experimental details were performed as previously described [17]. At 120 days post-injection, mice were sacrificed and tissues were examined macroscopically and microscopically for occurrence of metastases.

### Clonogenic assay

About 5 × 10^2 ^cells were added into each well of a six-well culture plate (three wells for each group). After incubation at 37°C for 14 days, the cells were washed twice with PBS and stained with 0.1% crystal violet solution. The number of colonies containing ≥20 cells was counted under a microscope.

### Subcutaneous tumorigenic assay

Subcutaneous administration of colon tumor cells was performed in the armpit area of nude mice. Approximately 1 × 10^6 ^cells were injected at each site. Mice were killed 30 days later, and tumorigenic incidence was assessed. The xenografts were excised for weight evaluation.

### Real-time RT-PCR

After FACS isolation, cells (3 × 10^5 ^cells per well) were cultured in six-well plates to 50% to 60% confluence. The treatment group was subjected to SDF-1 (Peprotech, Rocky Hill, NJ, USA) at a concentration of 100 ng/ml for 12 hours. The cells were collected to extract total cellular mRNA with Trizol reagent (Invitrogen, Carlsbad, CA, USA). Expression of mRNA was determined by real-time RT-PCR using SYBR Green Master Mix (Applied Biosystems, Foster City, CA, USA). Total sample RNA was normalized to endogenous GADPH mRNA. The sequences of primers used in this study are shown in Table 1. Thermal cycling conditions included an initial hold period at 95°C for four minutes; this was followed by a two-step PCR program of 95°C for 20 seconds and 72°C for 30 seconds repeated for 40 cycles on an Mx4000 system (Stratagene, La Jolla, CA, USA).

**Table 1:** Oligonucleotide sequences of primers used in real-time RT-PCR.

Gene	Sequence (5' → 3')	
E-cadherin	F	TGAAGGTGACAGAGCCTCTGGA
	R	TGGGTGAATTCGGGCTTGTT
Vimentin	F	TGGCCGACGCCATCAACACC
	R	CACCTCGACGCGGGCTTTGT
N-cadherin	F	GCGCGTGAAGGTTTGCCAGTG
	R	CCGGCGTTTCATCCATACCACAA
β-catenin	F	AGCCGACACCAAGAAGCAGAGATG
	R	CGGCGCTGGGTATCCTGATGT
Snail	F	CCTCCCTGTCAGATGAGGAC
	R	CCAGGCTGAGGTATTCCTTG
GAPDH	F	TGCCAAATATGATGACATCAAGAA
	R	GGAGTGGGTGTCGCTGTTG

### Boyden chamber invasion assay

A Boyden chamber was separated into two compartments by a polycarbonate membrane with an 8-mm pore, over which a thin layer of extracellular matrix (ECM) was dried. The ECM layer occluded membrane pores, blocking noninvasive cells from migrating. The Boyden chamber invasion assay was performed as previously described [31]. For the experiment that did not require SDF-1 treatment, 1 × 10^5 ^cancer cells in 200 μl of serum-free medium were added to the top chamber. McCoy's 5A Medium containing 10% FBS was added to the lower chamber. For the experiment subjected to SDF-1 treatment, both upper and lower chambers were filled with McCoy's 5A Medium containing 1% FBS for the control group, while SDF-1 at a concentration of 100 ng/ml was added to the lower chamber for the treatment group. After incubation for 48 hours, the noninvasive cells were removed with a cotton swab. The cells that had migrated through the membrane and adhered to the lower surface of the membrane were fixed with methanol for ten minutes and stained with crystal violet solution (0.1%). For quantification, the cells were counted using a microscope from five randomized fields at ×200 magnification.

### Transwell cell migration assays

Transwell cell migration assays were performed using a protocol similar to that used for the invasive assay described above. A Boyden chamber lacking a thin layer of ECM and a higher density of cells (2.5 × 10^5 ^cells) was used.

### Nude mouse hepatic metastasis assay

Cells (5 × 10^5^) were intrasplenically administered, and five minutes later the spleen was resected and more experiments were performed as previously described [32]. The three groups for injection were: CD133^+^CXCR4^- ^cells; CD133^+^CXCR4^+ ^cells; and CD133^+^CXCR4^+ ^cells with AMD3100 (Sigma, St. Louis, MO, USA) administration. AMD3100 (2.5 mg/kg) or PBS was intraperitoneally administered twice a day over 20 days. Mice were sacrificed 45 days later and livers were harvested to observe metastatic tumor formation.

### Statistical analysis

All of the *in vitro *experiments were repeated at least three times. The data were expressed as means ± SD. Statistical analysis was performed by Student's t test. The comparison of the incidence of tumor/metastasis formation by *in vivo *mouse models was performed with Fisher's exact test. For analyzing the association between CD133^+^CXCR4^+ ^cell content and various clinical factors, the 'age' as a continuous variable was expressed as mean (SD) and comparison between groups was done by using the Student's t-test. Categorical variables including gender, location, N status and M status were analyzed by Fisher's exact test and ranked variables including TNM (tumor-node-metastasis) staging, T status and grading were analyzed with the permutation test . The Kaplan-Meier method was used to estimate the medians for time-to-event parameters and to construct the survival curve. The equality of the two curves was compared by the permutation test. The permutation test was conducted by randomly permuting samples' labels (for example, high versus low CD133^+^CXCR4^+ ^cell content) and recomputing the two-sample statistic (for example, Log Rank χ2 for survival) for 50,000 times. The permutation *P *value was determined by the proportion of random permuted data sets which resulted in the same or more extreme statistic as observed in the actual data [33-35]. The permutation test was performed using SAS and all other analyses were performed using SPSS, version 17.0 (SPSS Inc, Chicago, Illinois) and tests were two-sided with a significance level <0.05 [36,37].

## Results

### CD133^+^CXCR4^+ ^cancer cell content is higher in hepatic metastasis than in human primary colorectal tumors

**Figure 1:**
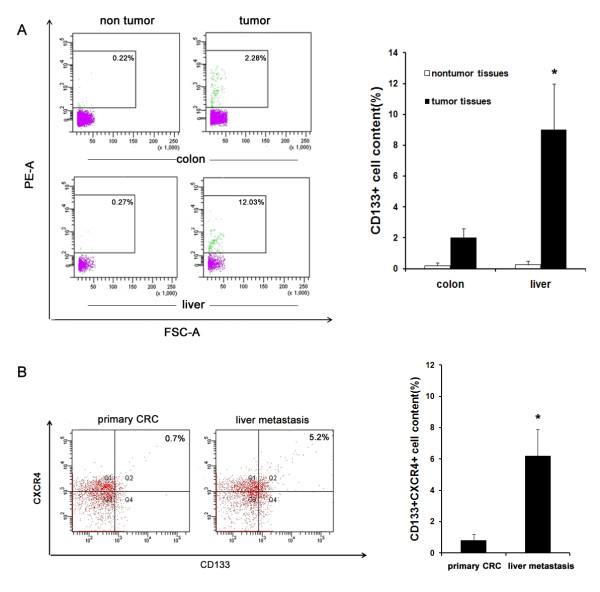
**CD133^+^CXCR4^+ ^cancer cell content is higher in hepatic metastasis than in human primary colorectal tumors**. (**A**) Specimens were digested, stained for CD133 and analyzed by flow cytometry to detect the CD133^+ ^subpopulation in primary and metastatic colorectal cancer as well as their corresponding normal tissues. A representative image is provided in the left panel. Quantification of flow cytometry data is presented in the right panel. (**B**) To assess the CD133^+^CXCR4^+ ^subpopulation in human specimens, primary and metastatic colorectal tumors were harvested, digested and double-stained for CD133 and CXCR4, then analyzed by flow cytometry. A representative image is provided in the left panel. Quantification of flow cytometry data is presented in the right panel (n = 9 analyzed patients).

We collected tissue samples from 29 patients with CRC (patient characteristics are given in Table 2). First, we aimed to identify CSCs with the widely recognized surface marker CD133 in primary CRCs, hepatic metastasis and their corresponding normal tissues. Flow cytometry analysis demonstrated that a rare population of CSCs was present in primary CRCs, while they were hardly detected in corresponding normal colorectal tissues. Furthermore, an increased number of CSCs were present in metastatic liver tumors, and the amount of CSCs in metastatic liver tumors was almost four times greater than those in primary colorectal tumors (Figure 1A). Next, because recent data have demonstrated that in some cancers there exists a subpopulation of migrating CSCs responsible for cancer metastasis and CXCR4 has been reported to be associated with the cancer cell metastasis phenotype, CD133^+^CXCR4^+ ^cells were also detected by flow cytometry. Results showed that the content of CD133^+^CXCR4^+ ^CSCs in metastatic liver tumors was more than seven times higher than that in primary CRCs (Figure 1B). These data demonstrate an enrichment of CD133^+^CXCR4^+ ^cells in metastatic cancers, which indicates that these cells may play a potential role in hepatic metastasis of CRC.

**Table 2:** Comparison of clinical characteristics between patients with low and high CD133^+^CXCR4^+ ^cell content.

	CD133^+^CXCR4^+ ^cell content			
			
Clinical factor	Low (number = 12)	High (number = 17)	Total (number = 29)	*P*
Age, years				
Mean(SD)	64(12.1)	59(11.4)		*P *= 0.27^a^
Gender				
Female	5	8	13	*P *= 0.28^b^
Male	7	9	16	
Location				
Colon	10	7	17	*P *= 0.02^b^
Rectum	2	10	12	
TNM stage^c^				
1	3	2	5	*P *= 0.02^d^
2	6	4	10	
3	2	3	5	
4	1	8	9	
T status				
1	3	4	7	*P *= 0.39^d^
2	2	3	5	
3	4	4	8	
4	3	6	9	
N status				
0	9	7	16	*P *= 0.06^b^
+	3	10	13	
M status				
0	11	9	20	*P *= 0.03^b^
1	1	8	9	
Grading				
1	3	2	5	*P *= 0.20^d^
2	4	6	10	
3	3	4	7	
4	2	5	7	
Median survival time (day)	710	489	580	*P *= 0.019^d^

^a^t test; ^b^Fisher's exact test; ^c^TNM is a cancer staging system and was assessed according to the 6^th ^edition of the UICC (International Union against Cancer); ^d^Permutation test. TNM, tumor-node-metastasis.

### CD133^+^CXCR4^+ ^colon cancer cells show higher migratory capacity than CD133^+^CXCR4^- ^cancer cells *in vitro*

**Figure 2:**
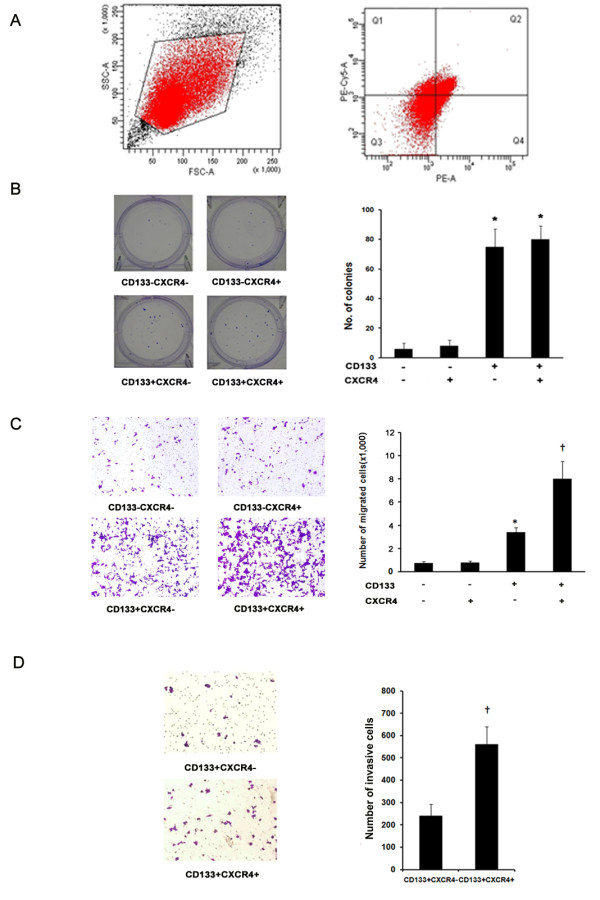
**CD133^+^CXCR4^+ ^colon cancer cells show higher migratory capacity than CD133^+^CXCR4^- ^cancer cells *in vitro***. (**A**) For *in vitro *and *in vivo *experiments, HCT116 cells were double-stained for CD133 and CXCR4. Four distinct phenotypic subpopulations, specifically CD133^-^CXCR4^-^, CD133^-^CXCR4^+^, CD133^+^CXCR4^- ^and CD133^+^CXCR4^+^, were isolated. (**B**) Clonogenic ability of the four phenotypic subpopulations was examined using clonogenic assay. A representative photograph is provided in the left panel, and quantified data of three independent experiments are shown in the right panel. (**C**) Transwell cell migration assays were performed to compare the migratory capacities of different phenotypic subpopulations. A representative photograph is shown in the left panel. Quantification of three independent experiments is shown in the right panel. (**D**) Boyden chamber invasion assays were performed to compare the invasive capacities of CD133^+^CXCR4^+ ^cells with CD133^+^CXCR4^- ^cells. A representative photograph is shown in the left panel. Quantification of three independent experiments is shown in the right panel. Bars represent the mean ± SD of invasive cells. (*P <0.01 compared with the CD133^-^CXCR4^- ^group, ^†^*P *<0.01 compared with the CD133^+^CXCR4^- ^group; ×200 magnification).

As Figure 1 showed that CD133^+^CXCR4^+^cells were increased in hepatic metastasis, in order to investigate the underlying mechanism of the phenomenon, we employed the human colon cancer cell line HCT-116 for *in vitro *and *in vivo *studies. Representative staining of CD133 and CXCR4 *via *flow cytometry is shown in Figure 2A. Four subgroups of cells were isolated using a high-speed FlowAria (Becton Dickinson) including CD133^-^CXCR4^-^; CD133^-^CXCR4^+^; CD133^+^CXCR4^-^; and CD133^+^CXCR4^+ ^subgroups. We performed clonogenic assays to detect the clonogenic capacity of the four phenotypic subpopulations. As shown in Figure 2B, much lower percentages of CD133^-^CXCR4^- ^and CD133^-^CXCR4^+ ^cells could form clones compared with CD133^+^CXCR4^- ^and CD133^+^CXCR4^+ ^cells. However, there was no significant difference in clone number between the CD133^-^CXCR4^- ^and CD133^-^CXCR4^+ ^groups, and between the CD133^+^CXCR4^- ^and CD133^+^CXCR4^+ ^groups. Next we performed transwell migration and invasion assays to compare the migratory and invasive capacities between CD133^+^CXCR4^- ^and CD133^+^CXCR4^+ ^cells. Our results showed that the numbers of migratory and invasive cells in the lower chamber of the CD133^+^CXCR4^+ ^group were greater than those in the CD133^+^CXCR4^- ^group (Figure 2C and 2D).

### CD133^+^CXCR4^+ ^colon cancer cells have higher metastatic potential in the nude mice model

**Figure 3:**
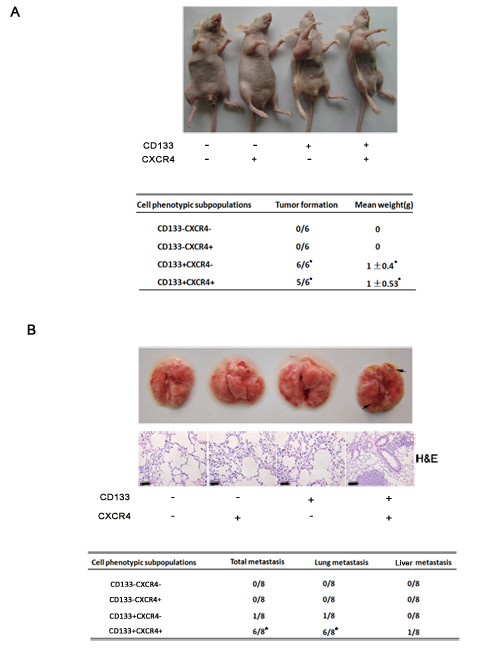
**CD133^+^CXCR4^+ ^colon cancer cells show higher migratory capacity than CD133^+^CXCR4^- ^cancer cells *in vivo***. (**A**) To assess the tumorigenic capacity of the four phenotypic subpopulations, cells were injected subcutaneously into nude mice. At 30 days post-injection, mice were killed and xenografts excised for evaluation. A representative photograph of four mice from four groups is shown in the upper panel. The tumorigenesis data from all groups are shown in the lower panel. Data represent the mean ± SD of tumor weight. (**B**) Standard tail vein metastatic assays were used to confirm that CD133^+^CXCR4^+ ^cells were responsible for metastatic cancer formation. Four phenotypic subpopulations isolated from HCT116 cells were injected into the tail veins of nude mice. After 120 days, the mice were sacrificed, and livers and lungs were harvested to observe metastatic tumor formation. A representative photograph of harvested lungs from the four groups is shown in the upper panel, with corresponding hematoxylin and eosin staining of metastatic lung tumor tissue. Arrows indicate metastatic lung nodules. The metastatic status of four groups is shown in the lower table. Comparisons between each group were made by Fisher's exact test or Student's *t*-test (*P <0.05 compared with the CD133^+^CXCR4^- ^group).

Tumorigenic and standard tail vein metastatic assays were employed to validate the *in vitro *findings reported above. Tumorigenic assays showed that CD133^-^CXCR4^- ^and CD133^-^CXCR4^+ ^cells were unable to develop into tumors in all six mice. The CD133^+^CXCR4^- ^subpopulation led to xenograft growth in all six mice, and in five of six mice for the CD133^+^CXCR4^+ ^cells (Figure 3A). Our results demonstrated that the status of CXCR4 expression does not result in different clonogenic and tumorigenic abilities for HCT116 colon cancer cells. CD133 can be regarded as an effective marker for colon CSCs. Standard tail vein metastatic assay was performed, and the four phenotypic subpopulations were injected into the tail veins of nude mice. Nude mice were sacrificed 120 days later, with liver and lung metastasis observed. As shown in Figure 3B, CD133^-^CXCR4^- ^cells and CD133^-^CXCR4^+ ^cells failed to form any metastasis in nude mice. Although CD133^+^CXCR4^- ^cells could form lung metastasis in one of eight mice, the metastatic frequency was much lower than that of CD133^+^CXCR4^+ ^cells, which resulted in lung and/or liver metastasis in six out of eight mice. These results suggest that CD133^+^CXCR4^+ ^cells represent a subpopulation in CSCs with high migratory capacity *in vitro *and *in vivo *in colon cancer cells.

### EMT contributes to high metastatic capacity of CD133^+^CXCR4^+ ^colon cancer cells

**Figure 4:**
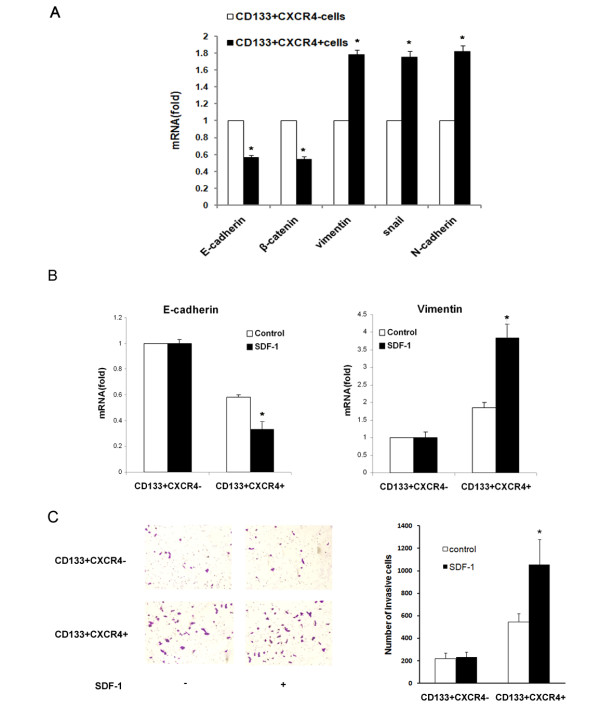
**EMT contributes to high metastatic capacity of CD133^+^CXCR4^+ ^colon cancer cells**. (**A**) Expression levels of mRNAs encoding E-cadherin, β-catenin, vimentin, Snail, and N-cadherin in CD133^+^CXCR4^+ ^cells and CD133^+^CXCR4^- ^cells, as determined by real-time RT-PCR. GAPDH mRNA was used to normalize the variability in template loading. The data are reported as mean ± SD. (*P <0.05; **P <0.01 compared with the CD133^+^CXCR4^- ^group). (**B**) Real-time RT-PCR was performed to determine the mRNA expression levels of E-cadherin (left panel) and vimentin (right panel) in CD133^+^CXCR4^- ^and CD133^+^CXCR4^+ ^cells with or without SDF-1 treatment. GAPDH mRNA was used to normalize the variability in template loading. The data are reported as mean ± SD. (*P <0.05 compared with the control CD133^+^CXCR4^- ^group). (**C**) A Boyden chamber assay was performed to compare the invasive capacities of CD133^+^CXCR4^- ^and CD133^+^CXCR4^+ ^cells with or without SDF-1 treatment. A representative photograph is shown in the left panel. Quantification of three independent experiments is shown in the right panel. Bars represent the mean ± SD of invasive cells. (*P <0.05 compared with the control CD133^+^CXCR4^+^group). EMT, epithelial-mesenchylal transition; SDF-1, stromal cell-derived factor-1.

Recent studies have shown that EMT is essential for the invasive and metastatic activity of human cancers [38]. We evaluated the mRNA levels of phenotypic EMT markers and regulatory factors including E-cadherin, β-catenin, vimentin, snail and N-cadherin in HCT116-derived CD133^+^CXCR4^- ^and CD133^+^CXCR4^+ ^cells by real-time RT-PCR. The expression of E-cadherin and β-catenin was down-regulated in CD133^+^CXCR4^+ ^cells compared with CD133^+^CXCR4^- ^cells. Vimentin, snail and N-cadherin were upregulated in the CD133^+^CXCR4^+ ^subpopulation (Figure 4A). This finding suggests that the metastatic activity of CD133^+^CXCR4^+ ^cells is partly attributable to the metastatic phenotype conferred by EMT.

The above data indicated that the CSC subpopulation expressing CXCR4 contribute to the metastasis of colon cancer. To detect whether the SDF-1 treatment could further induce the occurrence of EMT in CD133^+^CXCR4^+ ^cells, real-time RT-PCR was performed to examine the expression of E-cadherin and vimentin. As shown in Figure 4B, the mRNA expression of E-cadherin was down-regulated in CD133^+^CXCR4^+ ^cells after treatment with SDF-1 and vimentin expression was upregulated. Changes in mRNA expression for E-cadherin and vimentin were not observed in CD133^+^CXCR4^- ^cells after SDF-1 treatment. The transwell invasion assay was performed to examine whether SDF-1 treatment could also enhance the invasive properties of the CD133^+^CXCR4^+ ^subpopulation rather than CD133^+^CXCR4^- ^cells. There was no significant difference in the number of invasive cells in the lower chamber between untreated- and SDF-1-treated cells in the CD133^+^CXCR4^- ^group, while SDF-1 treatment almost doubled the number of invasive cells in the CD133^+^CXCR4^+ ^group (Figure 4C). These findings suggest that the metastatic property of CD133^+^CXCR4^+ ^cells may be partly attributable to further induction of EMT by SDF-1.

**Figure 5:**
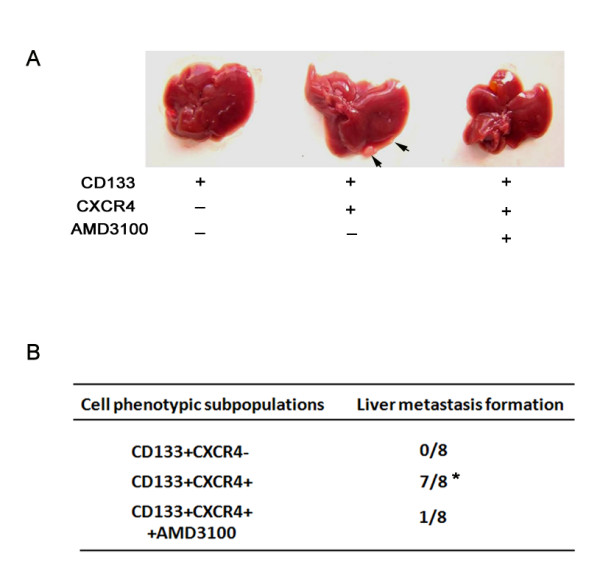
**Blockade of CXCR4 could inhibit hepatic metastases of colon cancer**. (**A**) The nude mouse hepatic metastasis model was employed to determine the effect of blocking the SDF-1/CXCR4 axis with AMD3100, a CXCR4 specific antagonist, on hepatic metastases. Cells were intra-splenically administered. The three groups were: CD133^+^CXCR4^-^; CD133^+^CXCR4^+^; and CD133^+^CXCR4^+ ^cells with continuous intraperitoneal administration of AMD3100. Mice were sacrificed 45 days later, and livers were harvested to observe metastatic tumor formation. A representative photograph of harvested livers is shown. (**B**) The hepatic metastasis status of the three groups is shown in the table. Comparisons between each group were made using Fisher's exact test (*P <0.01 compared with the CD133^+^CXCR4^- ^group).

SDF-1 further induced the occurrence of EMT in CD133^+^CXCR4^+ ^cells, with a previous report demonstrating that inhibition of the SDF-1/CXCR4 axis could impede the invasive behavior of CXCR4-expressing cells *in vitro *and metastatic cancer formation *in vivo *[21]. We used nude mice hepatic metastasis assays to examine the effect of blocking CXCR4 with AMD3100 on hepatic metastasis of colon cancer. As shown in Figure 5, only the injection of CD133^+^CXCR4^+ ^cells into the spleens of nude mice resulted in hepatic metastasis 45 days later, while injection of CD133^+^CXCR4^- ^cells failed to result in the formation of metastatic liver tumors. Furthermore, application of AMD3100, a pharmacological CXCR4 receptor inhibitor, was able to suppress the metastatic tumor burden in nude mice (Figure 5). Our results indicated that the SDF-1/CXCR4 system might be a potential target for the effective treatment of hepatic metastasis of CRC.

### High CD133+CXCR4+ cell content is associated with poor survival

**Figure 6:**
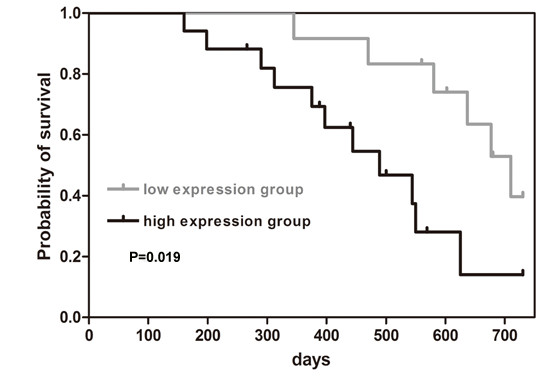
**High CD133^+^CXCR4^+ ^cell content is associated with poor survival**. Patients with high CD133^+^CXCR4^+ ^cell contents showed a significantly reduced two-year survival rate compared with patients with low CD133^+^CXCR4^+ ^cell contents using Kaplan-Meier survival curves (*P *= 0.019, permutation test based on 50,000 permutations).

We collected colorectal tumor specimens from 29 patients and determined the content of the CD133^+^CXCR4^+ ^subpopulation using flow cytometry. Patients were classified as having high or low CD133^+^CXCR4^+ ^cell contents according to the average CD133^+^CXCR4^+ ^cell percentage. The association of CD133^+^CXCR4^+ ^cell content with various clinical characteristics was determined by corresponding statistical methods (Table 2). A high CD133^+^CXCR4^+ ^cell content was significantly correlated with rectal tumors when compared with colon cancer (*P *= 0.02), high TNM stages (*P *= 0.02), and distant metastases as indicated by M status (*P *= 0.03). There was no significant association between high CD133^+^CXCR4^+ ^cell content and patient age, gender, T status, N status and tumor grade. With overall median survival time being 580 days, the median survival time for patients with high CD133^+^CXCR4^+ ^cell content was 489 days, and 710 days for patients with low CD133^+^CXCR4^+ ^cell content by the Kaplan-Meier method, indicating that patients with high CD133^+^CXCR4^+ ^cell content had decreased two-year survival (*P*-value for the permutation test was 0.019 based on 50,000 permutations; Figure 6).

## Discussion

CD133 has been used as a marker of tumor-initiating cells in neural cancers and is also generally accepted as a CSC marker for colon cancer [10-12]. However, there are some reports suggesting that CD133^+ ^cancer cells are not a true representation of CSCs in colon cancer [39,40]. We found that CD133^+ ^colon cancer cells isolated from the HCT116 cell line had a greater clonogenic and tumorigenic ability than CD133^- ^cells irrespective of CXCR4 expression. The *in vitro *and *in vivo *assays lend credence to the viewpoint that CD133 could be a marker for colon cancer tumor-initiating cells.

In 2005, Brabletz *et al*. proposed the concept that there are two forms of CSCs in tumor progression, namely stationary CSCs and migratory CSCs [13]. Hermann and colleagues published data supporting the existence of these two distinct subsets in CD133^+ ^pancreatic CSCs. The CSCs co-expressing CXCR4 were cancer cells with a migratory and invasive phenotype in pancreatic cancer [21]. In specimens from CRC patients,Pang *et al*. demonstrated the existence of migratory CSCs with the CD26 surface antigen as a marker [41]. In this study, we determined that the percentage of CD133^+^CXCR4^+ ^cancer cells in metastatic liver tumors was nearly eight times higher than that in primary colorectal tumors, indicating enrichment of this CSC subpopulation in metastatic liver tumors and their potential involvement in CRC metastasis to the liver. Transwell migration and invasion assay results indicated that the CD133+CXCR4+ subpopulation had higher migratory and invasive capacities *in vitro*. Consistent results were obtained by the standard tail vein metastatic assay *in vivo*. This indicated that CD133^+^CXCR4^+ ^cancer cells are a subpopulation of CSCs with a metastatic phenotype. To evaluate the metastatic capacity of different subpopulations, we employed the tail vein metastasis model, which is also known as the experimental metastasis model. The limitation of this model lies in the fact that it cannot reflect the complete metastatic process as does the spontaneous metastasis model in which the tumor cells are injected into the liver and allowed to first form a primary tumor. The complete metastasis cascade includes the following steps: escape of cells from the primary tumor, entry of cells into the lymphatic or blood circulation (intravasation), survival and transport in circulation, escape of cells from circulation (extravasation), and growth of cells to form secondary tumors in a new organ environment [42]. However, the tail vein metastasis model is able to mimic the extravasation of cancer cells from blood vessels in the target organ which is regarded as a critical step in the metastatic process[43]. Therefore, as in many studies[17,44,45], it is sufficient to use this model for the comparison of metastatic capacity among different groups.

EMT results in morphological and molecular changes that occur when epithelial cells lose their characteristics and gain mesenchymal properties. The expression of mesenchymal markers, such as N-cadherin and vimentin, and the loss of E-cadherin are key molecular events of EMT. Transcription factors, such as Snail and Twist, bind to consensus E-box sequences in the E-cadherin gene promoter and down-regulate E-cadherin transcription [46,47]. The association between EMT and CSC has been reported previously. Several studies have provided evidence showing that CSCs express EMT markers and that induction of EMT could convert epithelial cells into breast CSCs [27-30]. This demonstrates the essential role of EMT in CSCs acquiring invasive and metastatic phenotypes. We have proven our hypothesis that EMT is involved in the origin of migratory CSCs in colon cancer, using real-time RT-PCR to determine EMT-related gene expression. Pang *et al*. reported that EMT-like attributes contribute to the invasive phenotype and metastatic capacity of the migratory subpopulation in CRCs [41]. This is in line with our findings that the corresponding alteration in mRNA expression levels of EMT-related genes and higher migratory and invasive capacities have been observed in CD133^+^CXCR4^+ ^cancer cells. Furthermore, we found that treatment with SDF-1 could further induce the occurrence of EMT in CD133^+^CXCR4^+ ^cancer cells. The above data indicate that the CD133^+^CXCR4^+ ^subpopulation contributes to liver metastasis of colorectal cancer *via *EMT.

Consistent with our findings, Esther and colleagues demonstrated that transforming growth factor-β (TGF-β) induced the EMT process and de-differentiation in Fao rat hepatoma cells. This process coincided with upregulated CXCR4 expression and also sensitization of these cells to respond to SDF-1, which mediated migration [48]. Similar results were observed in oral squamous cell carcinoma [26,49]. However, the reason cancer cells that have undergone EMT have a higher expression of CXCR4 is far from clear. Exploring the origin of migratory CSCs warrants further research and requires integration of current tumor initiation and progression concepts, including CSC, EMT, accumulation of genetic alterations and the tumor environment as driving forces [13]. A deeper understanding of these factors could provide further insights into tumor biology.

The CSC hypothesis suggests that CSCs are a minority population that has the potential to self-renew, differentiate and regenerate a phenocopy of the original tumor. They would seem the most probable candidates that are resistant to chemotherapy, and they have been investigated previously [3,5,50-52]. Novel treatments targeting CSCs may result in the complete eradication of tumor growth, and furthermore, based on the migratory CSC theory, if treatment targeting migratory CSCs can be developed, it might be possible to prevent tumor metastasis. We hypothesized that blockade of the SDF-1/CXCR4 axis might suppress colon cancer metastasis to the liver, with the knowledge that the liver secretes high amounts of SDF-1 [53]. This is also in line with the theory that organs producing SDF-1 attract CXCR4^+ ^tumor cells and form metastatic tumors analogous to the directed homing of leukocytes. In our study, a nude mouse hepatic metastasis model was employed, and the results indicated that chemical inhibition of CXCR4 with AMD3100 could inhibit colon cancer metastasis to the liver. The anti-metastasis effect caused by the blockade of the SDF-1/CXCR4 axis is supported by another report [54]. This finding provides important clues for the development of a targeted therapy in the treatment of CRC.

To validate the above findings in *in vitro *experimental and in animal models, we carried out a prospective study to investigate whether CD133^+^CXCR4^+ ^cancer cell content was associated with disease progression and prognosis. Statistical analysis showed that high CD133^+^CXCR4^+ ^cell content is associated with poor 2-year survival of colorectal cancer patients. The clinical data provide evidence to support our hypothesis that double positive cancer cells might be involved in the metastatic process. Our data showed that cancer located in the rectum was associated with a high content of CD133^+^CXCR4^+ ^cancer cell compared with colon cancer. This might be due to higher CXCR4 expression in rectal cancer than in colon cancer[20], suggesting that the percentage of CD133^+^CXCR4^+ ^cancer cells in future studies should be investigated separately in colon and rectal cancer rather than in a mixed way.

## Conclusions

Taken together, our data demonstrate that CD133^+^CXCR4^+ ^cancer cells are possible migratory CSC subtypes in CRC. EMT is partly involved in these cells acquiring an invasive phenotype and metastatic behavior. Blockade of the SDF-1/CXCR4 axis could be developed for targeted therapy to control CRC metastasis.

## Abbreviations

CRC: colorectal cancer; CSC: cancer stem cell; EMT: epithelial-mesenchymal transition; PBS: phosphate buffered saline; RT-PCR: reverse transcriptase-polymerase chain reaction; SDF-1: stromal cell-derived factor-1; TNM: tumor-node-metastasis.

## Competing interests

The authors declare that they have no competing interests.

## Authors' contributions

Participated in the conception and design of the study and the critical revision of the manuscript for important intellectual content: LZ, LW, SZ, YJ, ZH. Performed the data collection and analysis: ST, HW, YW, RL, YY, XZ, XX, EY, YR, HL. Interpreted the data and produced the draft of the manuscript: SZ, ZH, ST, TL, HW, YW, RL, YY, XZ, XX, EY, YR, HL. Obtained funding for the study: LZ, LW. All authors read and approved the final version of the manuscript.

## Pre-publication history

The pre-publication history for this paper can be accessed here:

http://www.biomedcentral.com/1741-7015/10/85/prepub
